# Photonic–Electronic Modulated a-IGZO Synaptic Transistor with High Linearity Conductance Modulation and Energy-Efficient Multimodal Learning

**DOI:** 10.3390/mi16050517

**Published:** 2025-04-28

**Authors:** Zhidong Hou, Jinrong Shen, Yiming Zhong, Dongping Wu

**Affiliations:** School of Microelectronics, Fudan University, Shanghai 200433, China

**Keywords:** a-IGZO, synaptic transistor, artificial synapse, high linearity, neuromorphic application

## Abstract

Brain-inspired neuromorphic computing is expected to overcome the von Neumann bottleneck by eliminating the memory wall between processing and memory units. Nevertheless, critical challenges persist in synaptic device implementation, particularly regarding nonlinear/asymmetric conductance modulation and multilevel conductance states, which substantially impede the realization of high-performance neuromorphic hardware. This study demonstrates a novel advancement in photonic–electronic modulated synaptic devices through the development of an amorphous indium–gallium–zinc oxide (a-IGZO) synaptic transistor. The device demonstrates biological synaptic functionalities, including excitatory/inhibitory post-synaptic currents (EPSCs/IPSCs) and spike-timing-dependent plasticity, while achieving excellent conductance modulation characteristics (nonlinearity of 0.0095/−0.0115 and asymmetric ratio of 0.247) and successfully implementing Pavlovian associative learning paradigms. Notably, systematic neural network simulations employing the experimental parameters reveal a 93.8% recognition accuracy on the MNIST handwritten digit dataset. The a-IGZO synaptic transistor with photonic–electronic co-modulation serves as a potential critical building block for constructing neuromorphic architectures with human-brain efficiency.

## 1. Introduction

Neuromorphic computing, inspired by biological neural architectures, holds significant potential to circumvent the von Neumann bottleneck through in-memory processing, thereby addressing the fundamental limitations imposed by the memory wall [1,2,3,4,5]. The emulation of synaptic neurotransmission mechanisms constitutes a fundamental prerequisite for developing neuromorphic architectures [6,7]. Artificial synapse modulation can be divided into two types: Optical stimuli enable non-contact operation, high-speed broadband signal transmission, and low power consumption via minimized RC delays and electromagnetic interference [8]. But, they have faced a significant challenge in large-scale integrations. Electrical stimuli offer precise signal control and CMOS compatibility but face bandwidth–density trade-offs and higher energy losses [9]. Their complementary strengths highlight the potential for integrated optoelectronic architectures in energy-efficient neuromorphic computing.

The performance of neuromorphic computing systems fundamentally relies on the linearity and symmetry characteristics of their constituent synaptic devices, which determine signal processing accuracy and system robustness [10,11,12]. However, current research on memory-based neuromorphic devices primarily focuses on emulating responses to single-spike excitation/inhibition, paired-pulse facilitation, and specific biological behaviors [13,14,15]. Studies aimed at improving critical parameters for neuromorphic computing, such as multilevel conductance states, linearity, and the symmetric ratio, remain insufficient, limiting the advancement of neuromorphic applications [10,16,17].

Amorphous indium–gallium–zinc oxide (a-IGZO) has garnered attention due to its uniform and isotropic characteristics, high carrier mobility, and capability for room-temperature fabrication [18,19,20]. Furthermore, Fowler–Nordheim (F-N) tunneling enables the accurate modulation of electron injection to and extraction from the floating gate during each operation, allowing the meticulous adjustment of channel conductance through precise pulsed control [21,22,23]. However, previously reported a-IGZO synapse transistors do not simultaneously achieve both the biomimetic shape of the post-synaptic current (especially the inhibitory post-synaptic currents) and high linearity under multiple pulse conditions [24,25,26,27,28]. The substantial noise in the post-synaptic current poses a critical barrier to the development and practical deployment of neuromorphic computing systems.

Here, a molybdenum (Mo)-engineered charge-trapping a-IGZO synaptic transistor with photonic–electronic co-modulation is proposed. The Mo floating gate possesses the ability to store a large amount of charge, allowing the channel to exist in multilevel conductance states and ensuring uniform changes in post-synaptic currents after each equivalent stimulation. The a-IGZO synaptic transistor not only effectively realizes excitatory/inhibitory post-synaptic currents (EPSCs/IPSCs), but also exhibits outstanding properties in long-term potentiation/depression (LTP/LTD), including its multilevel conductance states (100/100), high linearity (nonlinear, NL, 0.0095/−0.0115), and asymmetric ratio (AR, 0.247). Furthermore, a bio-inspired classical conditioning paradigm was successfully emulated through optoelectronic co-stimulation, demonstrating spatiotemporal coordination of photonic input (365 nm) and electronic reinforcement signals (100 μs) in neuromorphic circuits. Leveraging device-level long-term plasticity, the implemented neuromorphic synaptic network achieved a classification accuracy of 93.2% on the simulated MNIST digit classification benchmark.

## 2. Experiment

A heavily p-doped Si substrate with a 100 nm thermal SiO_2_ layer was cleaned using acetone, isopropyl alcohol, and deionized water. First, a Mo bottom floating gate layer was deposited by DC sputtering. Next, an Al_2_O_3_ tunneling layer was deposited by atomic layer deposition (trimethylaluminum (TMA, Al(CH_3_)_3_) served as the aluminum precursor, oxygen plasma was employed as the oxygen source, 200 °C, 0.1 nm/cycle). An a-IGZO film was then deposited by RF sputtering using an IGZO target with an In/Ga/Zn/O atomic ratio of 1:1:1:4 (110 W, 0.4 Pa, argon, 20 °C, 0.8 nm/min). Finally, 50 nm tungsten (W) source and drain electrodes were deposited by DC sputtering. The channel length and width of the device were both 10 µm. All patterns were defined using standard lithography techniques. Cross-sectional images were obtained using a Thermo Scientific Themis Z aberration-corrected transmission electron microscope (TEM, Thermo Fisher Scientific, Waltham, MA, USA). Specimens for TEM measurement were prepared by focused ion beam (FIB) milling with a Thermo Scientific Helios G4 HX (Thermo Fisher Scientific). EDS analysis data were obtained using the Super X FEI System in STEM mode. Electrical characterizations were conducted under ambient conditions (25 °C, 1 atm) using a Keysight B1500A semiconductor parameter analyzer in a dark environment (Keysight, Santa Rosa, CA, USA).

## 3. Results and Discussion

**Figure 1:**
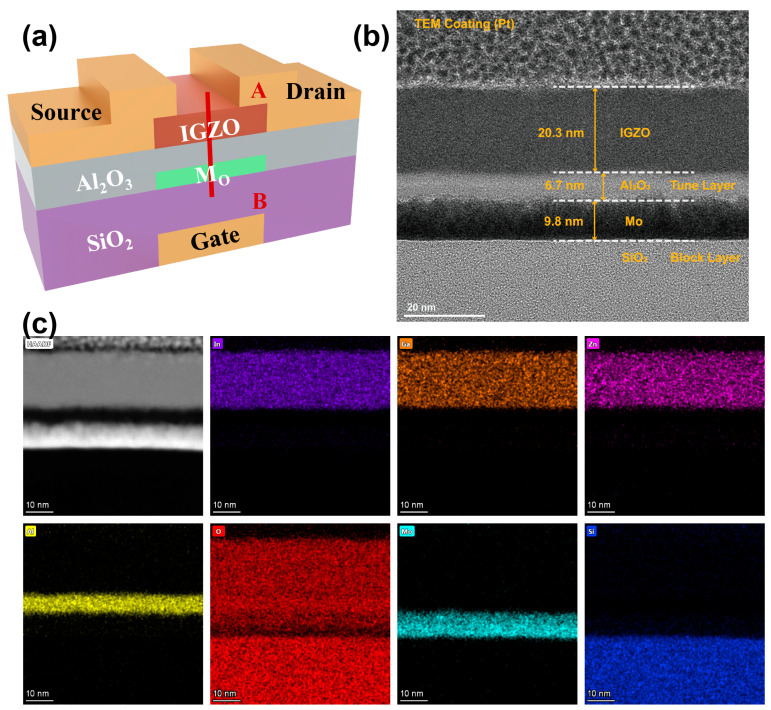
(**a**) Schematic illustration of the a-IGZO artificial synapse. (**b**) TEM and (**c**) EDS mapping image of the synapse (along the A-B line).

**Figure 2:**
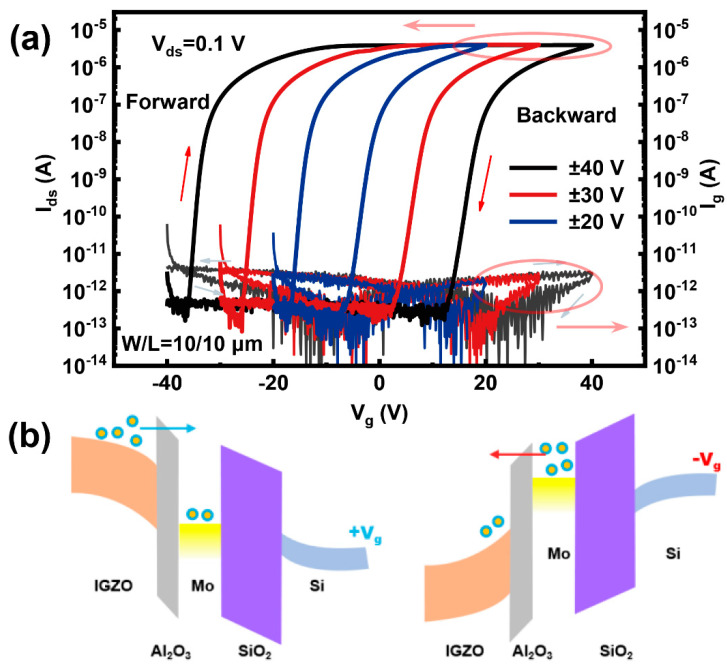
(**a**) Double-sweep transfer curves for three gate voltage ranges (±20, ±30, and ±40 V). (**b**) The schematic energy band diagram under bias.

The schematic diagram of the fabricated a-IGZO synapse and the cross-sectional TEM image along the A-B line are shown in Figure 1a and Figure 1b, respectively. This device employs a heavily p-doped Si substrate, which also serves as the gate electrode, an a-IGZO layer as the channel, an Al_2_O_3_ layer as the tunneling oxide, a Mo layer for charge trapping, and a 100 nm SiO_2_ layer as the blocking oxide. The cross-sectional TEM image and EDS mapping show that all layers exhibit distinct boundaries and a uniform deposition (Figure 1b,c). The device demonstrates a clear transfer curve with a large memory window, low leakage through the gate current (I_g_, ~1 pA), and a high on/off ratio exceeding 10^7^ (Figure 2a). As the gate voltage (V_g_) sweep range increases, the double-sweep transfer curve displays larger clockwise hysteresis due to threshold voltage (Vth) shifts resulting from the charge-trapping process. Figure 2b schematically illustrates the energy band diagrams under applied bias, providing a description of the charge transport and trapping mechanisms within the device.

**Figure 3:**
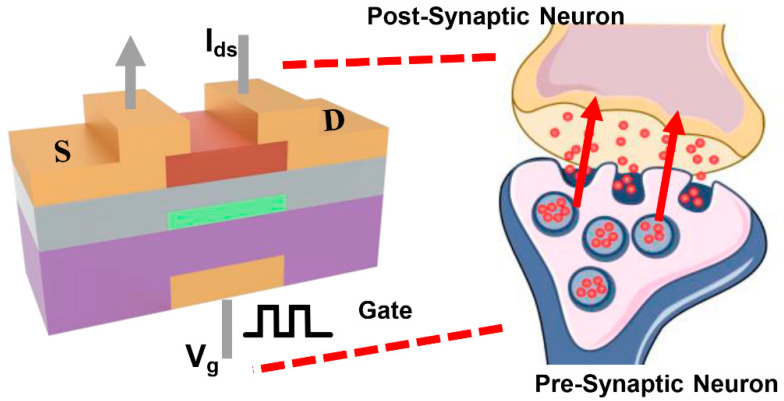
The schematic of the a-IGZO synapse and a biological synapse. The gate terminal functions as the pre-synaptic neuron, responsible for delivering input signals. The channel conductance represents the synaptic weight, analogous to the strength of the connection between neurons, and is modulated by applied gate voltage pulses. The source and drain terminals act as the post-synaptic neuron, receiving the signal in the form of changes in current.

**Figure 4:**
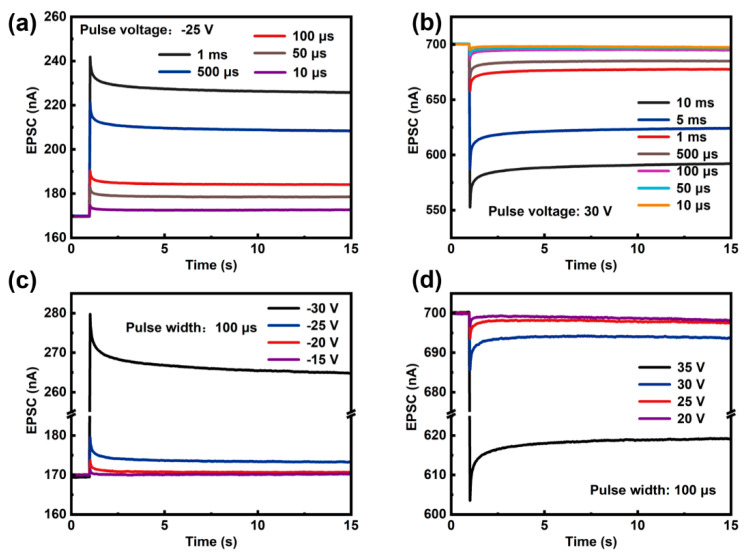
(**a**) EPSC and (**b**) IPSC responses triggered by pulses of varying widths. (**c**) EPSC and (**d**) IPSC responses triggered by pulses of varying voltages. The reading voltage of the synaptic device was 0.1 V.

In the human brain, synapses form connections between neurons and facilitate the transmission of electrical signals from pre-synaptic to post-synaptic neurons (Figure 3) [29]. The efficacy of neuronal communication, collectively termed synaptic weight, is dynamically regulated through neuro-modulatory pathways. This dynamic modulation system constitutes a substrate for adaptive neural computation and information storage in biological neural networks [30]. Artificial synapses typically quantify these two characteristics using the magnitude of EPSC/IPSC. In the a-IGZO artificial synapse, the channel conductance state (I_ds_) is used as the synaptic weight, with its plasticity being modulated by an applied V_g_ pulse. Figure 4a–d present the pulse-voltage- and pulse-width-dependent EPSC/IPSC plasticity. For a single excitatory pulse, the EPSC increases as the pulse width or voltage increases, while the other parameter is held constant. This relationship also applies to the IPSC. Thus, the synaptic weight can be flexibly tuned by an electrical pulse, demonstrating that the plasticity of an a-IGZO synapse can be realized by the pulse stimulations.

**Figure 5:**
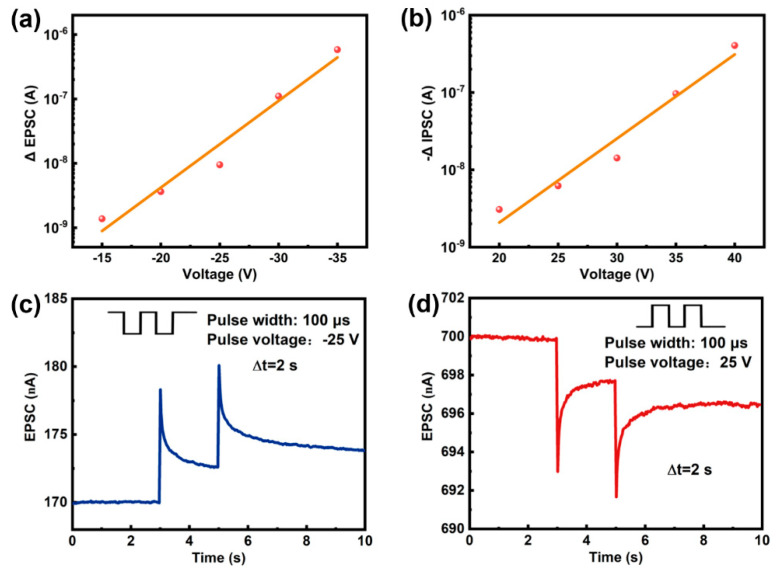
(**a**) The dependence of the EPSC and (**b**) IPSC on the pulse voltage. (**c**) PPF and (**d**) PPD performance of the a-IGZO synapse.

As illustrated in Figure 2b, when a sufficiently positive pulse is applied, a certain number of electrons in the IGZO tunnel through the Al_2_O_3_ layer and become trapped in the Mo charge-trapping layer. These trapped electrons cannot return, leading to a decrease in I_ds_, which corresponds to the IPSC. Conversely, a sufficiently negative pulse transfers electrons from the Mo layer to the IGZO, resulting in an increase in I_ds_, corresponding to the EPSC. The steady-state F-N tunneling current density exhibits an exponential dependence on the electric field (J ∝ E^2^exp(−B/E), where E is the internal electric field strength in the oxide layer and B is a constant related to the barrier height). As shown in Figure 5a,b, the post-synaptic current shows an exponential relationship with voltage, which confirms that charges in the proposed artificial synapse are injected into the trapping layer through F-N tunneling.

Paired-pulse facilitation (PPF) and paired-pulse depression (PPD) are critical forms of short-term synaptic plasticity involved in learning and memory processes in the human brain [31,32]. In biological synapses, such behavior is closely associated with short-term plasticity and plays a vital role in sensory processes like pain perception. The artificial synapse successfully replicates PPF/PPD, as demonstrated in Figure 5c,d. The change in the amount of charge in the Mo floating gate accumulates as the number of pulses increases. Consequently, the post-synaptic current generated by the second presynaptic spike surpasses that induced by the first.

**Figure 6:**
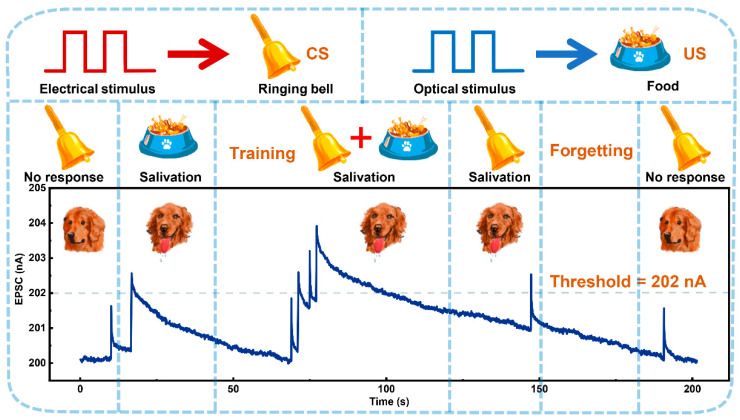
Simulation of Pavlovian conditioned reflex using the photonic–electronic co-modulated a-IGZO artificial synapse. The optical stimuli (unconditioned stimulus, US, 365 nm) had an intensity of 265 μW/cm^2^ with a duration of 250 ms, while the electrical stimulus (conditioned stimulus, CS) was applied at −17 V for 100 μs.

The metastable carrier-trapping dynamics underlying the persistent photoconductivity effect in amorphous IGZO channels enable precise emulation of light-stimulated synaptic potentiation behaviors at the device level [33,34]. To demonstrate the application of the proposed photonic–electronic co-modulated artificial synapse in complex associative learning patterns, it was used to simulate the Pavlovian conditioned reflex [16,35]. Both optical and electrical stimuli were employed, with the optical stimuli representing food as the unconditioned stimulus (US) and the electrical stimuli representing the ringing of a bell as the conditioned stimulus (CS). An EPSC value of 202 nA was defined as the threshold for triggering the salivation response. As shown in Figure 6, the initial electrical stimulus (CS) did not trigger a response, whereas the optical stimulus (US) successfully did. During the training stage, both stimuli were applied simultaneously twice to establish an association between the bell ringing and the salivation response. After conditioning, the EPSC induced by a single electrical stimulus exceeded the threshold, effectively triggering the conditioned salivation response. As the EPSC decayed further, the rise induced by the electrical stimulus alone fell below the threshold, failing to initiate a response and simulating the forgetting process of the conditioned response. This demonstrates that the photonic–electronic co-modulated synapse successfully mimics the learning and forgetting behaviors in a Pavlovian conditioned reflex.

**Figure 7:**
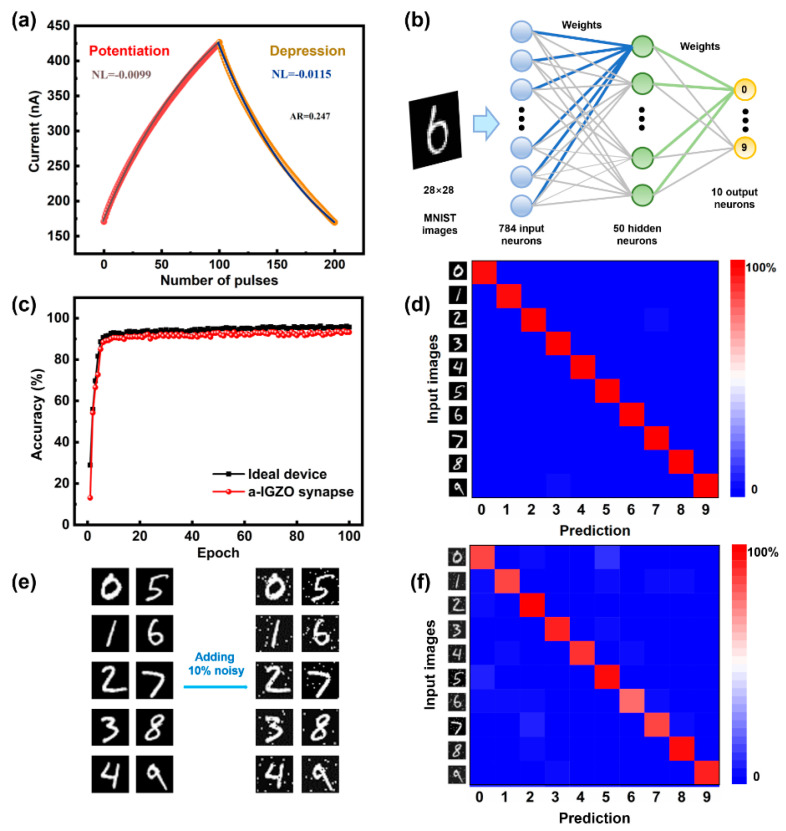
(**a**) Conductance states of the a-IGZO artificial synapse and the corresponding fitting curves. The LTP/LTD comprise 100 potentiation pulses (−20 V/100 μs) and 100 depression pulses (32 V/100 μs). (**b**) Illustration of a three-layer neural network, consisting of an input layer, a hidden layer, and an output layer. (**c**) Simulated pattern recognition accuracy as a function of training epochs of artificial synapses in comparison with the ideal case. (**d**) The confusion matrix comparing the desired and predicted values after 60 training epochs. (**e**) Examples of MNIST handwritten digit images before and after adding 10% noise. (**f**) Confusion matrix for handwritten digit recognition after noise addition. The maximum values appear along the diagonal, corresponding to successful digit identification.

Long-term plasticity, including both LTP and LTD, is essential for learning and memory functions in biological nervous systems [36]. Generally, when an artificial synapse exhibits higher linearity and symmetry in conductance regulation, the neuromorphic computing it composes will show better performance. As shown in Figure 7a, the LTP/LTD characteristics of the IGZO synapse were measured and exhibited remarkable properties, including 100 conductance states in both potentiation and depression, good linearity with NL coefficients of 0.0099 and −0.0115, and an AR of 0.247, as calculated using the formulas in the reference [36]. These evaluation parameters significantly surpass those of other similar devices [37,38,39]. The a-IGZO artificial synapse consumes between 1.7 and 4.3 pJ of energy per spike during weight modulation, following the calculation method in reference [40].

Moreover, multilevel conductance states with high linearity and symmetric conductance updates have been established as essential features for optimizing neuromorphic computing performance. The image recognition accuracy was evaluated using MNIST handwritten digit images to demonstrate the potential of this synapse in neuromorphic computing. The artificial neural network simulation employed a three-layer neural network with 784 input neurons, 50 hidden neurons, and 10 output neurons, as shown in Figure 7b. Figure 7c illustrates the recognition accuracy of the neuromorphic network incorporating IGZO synapses. The accuracy of the network improved to 90.0% after 20 training epochs and then reached 93.8% and stabilized there after 60 training epochs. Notably, the theoretical maximum accuracy for digit recognition using the back-propagation algorithm is 96.8% [27]. The confusion matrix comparing the desired and predicted values after 60 training epochs is presented in Figure 7d, highlighting the effectiveness of IGZO synapses in enhancing recognition accuracy. After the addition of 10% noise-level pixels to MNIST test images, the recognition accuracy of the synaptic-based ANNs remains above 83% (Figure 7e,f). The above-mentioned results suggest that the proposed synapse is a promising candidate for constructing neuromorphic computing networks.

## 4. Conclusions

In summary, the proposed a-IGZO photonic–electronic co-modulated synapse successfully simulates synaptic plasticity (including good linearity, excellent symmetry, and a high number of conductance states) and effectively mimics biological conditioned reflex behaviors. The neuromorphic network based on this synapse achieved a notable accuracy of 93.8% in handwritten digit recognition. The novel photonic–electronic co-modulated a-IGZO synaptic transistor demonstrates significant potential as a core functional element in neuromorphic computing architectures, effectively bridging optoelectronic modulation with biologically inspired synaptic emulation capabilities.

## Data Availability

The original contributions presented in the study are included in the article, further inquiries can be directed to the corresponding author.
